# Relating Doses of Contrast Agent Administered to TIC and Semi-Quantitative Parameters on DCE-MRI: Based on a Murine Breast Tumor Model

**DOI:** 10.1371/journal.pone.0149279

**Published:** 2016-02-22

**Authors:** Menglin Wu, Li Lu, Qi Zhang, Qi Guo, Feixiang Zhao, Tongwei Li, Xuening Zhang

**Affiliations:** 1 Radiology department, Second Hospital of Tianjin Medical University, Hexi District, Tianjin, China; 2 Department of General surgery, Tianjin Medical University General Hospital, Heping District, Tianjin, China; University of Chicago, UNITED STATES

## Abstract

**Objective:**

To explore the changes in the time-signal intensity curve(TIC) type and semi-quantitative parameters of dynamic contrast-enhanced(DCE)imaging in relation to variations in the contrast agent(CA) dosage in the Walker 256 murine breast tumor model, and to determine the appropriate parameters for the evaluation ofneoadjuvantchemotherapy(NAC)response.

**Materials and Methods:**

Walker 256 breast tumor models were established in 21 rats, which were randomly divided into three groups of7rats each. Routine scanning and DCE-magnetic resonance imaging (MRI) of the rats were performed using a 7T MR scanner. The three groups of rats were administered different dosages of the CA0.2mmol/kg, 0.3mmol/kg, and 0.5mmol/kg, respectively; and the corresponding TICs the semi-quantitative parameters were calculated and compared among the three groups.

**Results:**

The TICs were not influenced by the CA dosage and presented a washout pattern in all of the tumors evaluated and weren’t influenced by the CA dose. The values of the initial enhancement percentage(E_first_), initial enhancement velocity(V_first_), maximum signal(S_max_), maximum enhancement percentage(E_max_), washout percentage(E_wash_), and signal enhancement ratio(SER) showed statistically significant differences among the three groups (*F* = 16.952, *p* = 0.001; *F* = 69.483, *p*<0.001; *F* = 54.838, *p*<0.001; *F* = 12.510, *p* = 0.003; *F* = 5.248, *p* = 0.031; *F* = 9.733, *p* = 0.006, respectively). However, the values of the time to peak(T_peak_), maximum enhancement velocity(V_max_), and washout velocity(V_wash_)did not differ significantly among the three dosage groups (*F* = 0.065, *p* = 0.937; *F* = 1.505, *p* = 0.273; *χ2* = 1.423, *p* = 0.319, respectively); the washout slope(Slope_wash_), too, was uninfluenced by the dosage(*F* = 1.654, *p* = 0.244).

**Conclusion:**

The CA dosage didn’t affect the TIC type, T_peak_, V_max_, V_wash_ or Slope_wash_. These dose-independent parameters as well as the TIC type might be more useful for monitoring the NAC response because they allow the comparisons of the DCE data obtained using different CA dosages.

## Introduction

Breast cancer is the most common cancer prevalent in women [1]. According to the American Cancer Society report, 2013 [2], breast cancer accounted for 30% of the malignancies in American women. Clinicians have focused on the early diagnosis of the disease, which is the key to successful treatment. Magnetic resonance imaging(MRI), particularly dynamic contrast-enhanced (DCE)-MRI, significantly improves the accuracy of the diagnosis of breast diseases [1]. The unique ability of this technique to reveal the hemodynamic characteristics of suspicious lesions helps distinguish benign diseases from malignant ones [3]. Furthermore, this application has been extended to other organs such as the ovary [4] and prostate [5].

Recently, DCE studies have emphasized on computer-aided diagnosis (CAD) [6] and predicting the response to neoadjuvant chemotherapy (NAC) responses or a combination of these two objectives [7–8] through the use of DCE parameters in CAD systems to test their ability to predict response [9]. The commonly used parameters can be divided into quantitative parameters (which have high temporal resolution and relatively low spatial resolution) and semi-quantitative parameters (which, conversely, have high spatial resolution and low temporal resolution). All of these parameters have been studied for their abilities to differentiate between diagnoses and predict the NAC responses. Although the quantitative parameters derived from pharmacokinetic models seem to be more precise compared to the time-signal intensity curve (TIC) based semi-quantitative parameters, the compromised spatial resolution, time-consuming nature of the study, and the necessity for expensive software limit their large-scale clinical applications. More and more researchers have found that the more practical semi-quantitative parameters have good relationships with the pharmacokinetic parameters [10–11], and that they might be useful as predictors of a good response to NAC or long-time survival [12].

However, there is no standardized DCE scanning process available as yet [13]. In cases where the imaging protocols are not exactly the same, discrepancies in theMR scanners, temporal resolutions, delayed-phases, magnetic field intensities, contrast agents (CAs), and CA dosages [14–16] could affect the related semi-quantitative parameters, which might, in turn, affect the results of the comparison of the pre and post-NAC paired parameters. In this research, we focused on evaluating the effect of the CA dosage on the semi-quantitative parameters by DCE scanning. Although the recommended dosage for breast MRI by the American College of Radiology is 0.1mmol/kg via a bolus injection followed by at least 10mL saline flush [17], in clinical practice, the actual dosage might be as high as 0.2 mmol/kg [18–19], particularly during the evaluation of NAC responses, since higher dosages might improve the conspicuity of the malignant lesions [20]. As an alternative to the use of a fixed dose, injection of a fixed volume of CA regardless of the patient's body weight is also performed. Jansen et al. [21] reported that a fixed-volume injection could result invariations in the CA dosage in most patients. In their study, the patients were divided into three groups according to the CA dosage(group 1,<0.122mmol/kg; group 2, 0.123–0.155 mmol/kg; group 3,>0.155 mmol/kg), and the results showed that the initial and peak enhancement percentages (E_first_ and E_max_, respectively) had a trend to increase when the dose was increased. It is worth noting that since the dosages used in their study were continuous data and divided into three groups without specific classifications, the results cannot be absolutely precise. In order to further investigate the changes in the semi-quantitative parameters because of differences in the CA dosages, we built a murine mammary carcinogenesis model and injected different dosages of CA (0.2 mmol/kg, 0.3 mmol/kg and 0.5 mmol/kg) for DCE scanning to clarify the effects on a number of semi-quantitative parameters.

## Materials and Methods

### Ethics statement

All animal care and experimental protocols followed the local animal ethics regulations and were approved by the Tianjin Medical University Animal Care and Use Committee. Female Wistar rats were purchased from the Animal Resource Center at the Institute of Radioactive Medicine, Chinese Academy of Medical Sciences(Tianjin, China) and maintained in a pathogen-free animal room with a controlled temperature at 22–24°C and humidity at 50–55% under a 12-hr light/dark cycle. All mice had free access to autoclaved food and fresh water. The physical conditions of the animals were monitored every day. The rats were sacrificed by isoflurane inhalation followed by cervical dislocation. All efforts were made to ameliorate the suffering of the animals.

### Cell culture and animal model preparation

The murine Walker 256 breast cancer cell line was obtained from the American Type Culture Collection (ATCC, Rockville, MD, USA). Walker 256 cells were cultured in RPMI 1640 medium supplemented with 10% fetal bovine serum and 2mM L-glutamine. The cells were incubated at 37°C in a mixture of 5% CO_2_ and 95% air. The cells were harvested during the logarithmic phase, and the concentration was adjusted to 1×10^7^ cells/mL. 1mL cell suspension was injected into the abdominal cavity of a female Wistar rat (weighing approximately 120g). After one week, the carcinomatous ascites was withdrawn, and the cell concentration was adjusted to 1×10^7^ cells/mL. Then, we injected 0.2mL medium/per rat(35 female Wistar rats weighing approximately 160g) subcutaneously in the region of the right groin. All the rats were housed in pathogen-free facilities and provided with rodent chow and tap water. Electronic digital calipers were used to measure the tumor size. The tumor volume wascalculated using the formula, volume = (length×width^2^)/2. None of the rats experienced difficulties with normal locomotion or access to food and water. After a week, the rats with tumors(tumor volume range: 0.0625–0.162 cm^3^) were subjected to further MR scanning. None of the rats became ill or died before MRI examination.

### MR data acquisition

The MR images were acquired using the Burker Pharmascan 7.0T MR scanner(7T, Pharmascan, Bruker, Germany) with a dedicated 4-channel volume coil. The twenty-one rats included in this study were divided into three groups according to the CA dosage to be injected. Each group included 7rats. The rats were anesthetized using a mixture of2% isoflurane and 98% oxygen. The respiratory rate, temperature, and electrocardiogram signals were monitored throughout the entire scanning process. The rats were placed on the scanning bed in the prone position. Routine MR images were acquired using T1-weighted imaging(T1WI) and T2-weighted imaging(T2WI) sequences were acquired for tumor localization and better visualization of the extra tumoral tissues: (1) T1WI repetition time (TR) = 500ms; echo time (TE) = 15ms; NEX = 2; slice thickness = 1.0mm; slice space = 0mm; FOV 5×5 cm; and matrix 256×256; (2) T2WI TR = 3000ms and TE = 45ms; the rest of the scanning parameters were the same as those used for T1WI. The parameters of the two-dimensional fast low angle shot(2D FLASH) sequence used to obtain 41 serial DCE-MR images at each of the three axial oriented planes were the same as those for the T1W and T2W sequences, except TR = 29ms, TE = 4.3ms, and flip angle (FA) = 30°, all three imaging scan planes were located at the center of the tumor. The DCE imaging sequence was repeated two times before the injection of the CA, following which, it was repeated 39 times beginning at 50s post-injection. The acquisition time for one serial dataset was 29s. Summary of MRI protocols is shown in Table 1. The contrast medium used in this experiment was gadopentetate dimeglumine (Gd-DTPA, Magnevist, Wayne, NJ), and the dosages were 0.2 mmol/kg for group1, 0.3 mmol/kg for group2, and 0.5 mmol/kg for group3, respectively. The CA was diluted with saline to a fixed volume at 0.4mL and delivered within 50 seconds via a tail-vein catheter.

**Table 1:** Summary of MRI protocols.

	TIWI	T2WI	2D FLASH
TR, ms	500	3000	29
TE, ms	15	45	4.3
NEX	2	2	2
Flip angle	0°	0°	30°
FOV, cm	5×5	5×5	5×5
Matrix	256×256	256×256	256×256
Number of phase encoding steps	256	256	256
Slice thickness, mm	1.0	1.0	1.0
Slice space, mm	0	0	0
Pattern of the k-space	Radical	Radical	Radical
Parallel imaging technique	No	No	No

### MR data analysis

**Fig 1:**
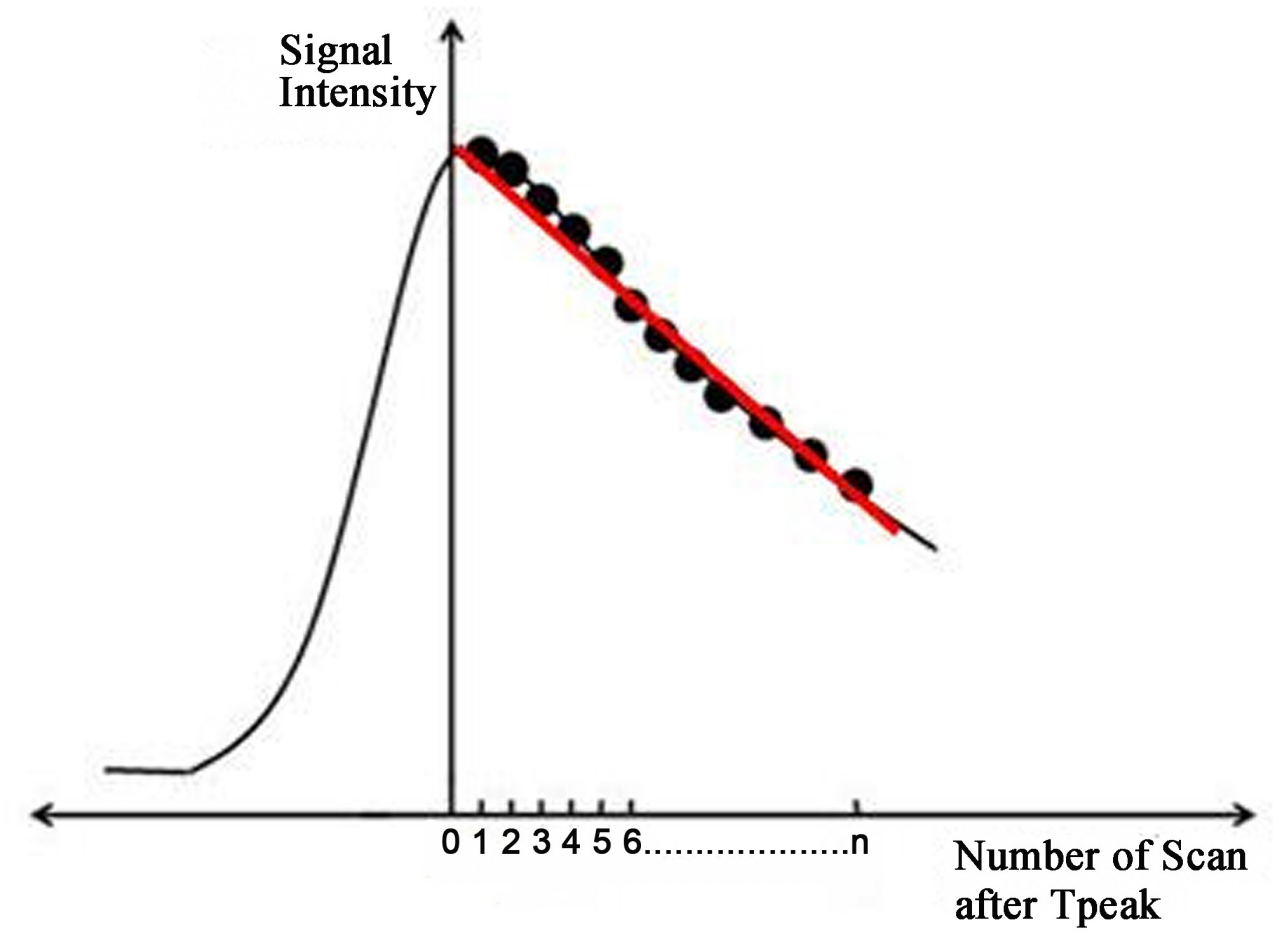
The red curve represents the fitted line of the effluent curve, and the slope of this fitted line is defined as Slope_wash_.

The tumor signal was detected by defining a region of interest (ROI) inside the tumor, while avoiding the areas of hemorrhage and necrosis; one ROI was defined per image. Using the SER software of the ADW 4.6 workstation (GE, USA), we obtained tumor signals at the exact same areas in all of the datasets. The average signal intensity of three ROIs in one dataset was recorded as the final signal of the tumor in that dataset (S_n_). Because the sequence was repeated two times before CA injection, the average of the signal intensities at six ROIs was recorded, the mean signal intensity(S_0_), corresponding to the signal before CA injection. For easy representation of the TIC, the number of scans before CA injection was indicated as 0, the number of the first scan post-CA injection was designated as 1, and the number of the last scan number of the entire DCE sequence was designated as39. The TIC was depicted by connecting all of the signal intensities according to the time order. Typically, TICs are divided into three categories: typeI, steady enhancement with a straight or curved time-signal intensity line; type II, plateau of signal intensity; type III, washout of the signal intensity [11]. Accordingly, we determined the type of TIC, and then we used the TIC to calculate the semi-quantitative parameters. The formulae or methods used for these calculationsare shown in Table 2 and Fig 1.

**Table 2:** Formulae or methods used for the calculation ofsemi-quantitative parameters evaluated in this study.

Parameter	Abbreviation	Formula/Method
Early enhancement parameters		
Initial enhancement percentage	E_first_	= (S_1_-S_0_)/S_0_
Initial enhancement velocity	V_fitst_	= (S_1_-S_0_)/T_1_
Peak parameters		
Maximum signal	S_max_	Maximum enhancement signal observed post-CA administration
Time to peak	T_peak_	Time (s) taken to achieve the maximum enhancement signal
Maximum enhancement percentage	E_max_	= (S_max_-S_0_)/S_0_
Maximum enhancement velocity	V_max_	= (S_max_-S_0_)/T_peak_
Washout parameters		
Washout percentage	E_wash_	= (S_max_-S_39_)/S_max_
Washout velocity	V_wash_	= (S_max_-S_39_)/(T_39_-T_peak_)
Signal enhancement ratio	SER	= (S_1_-S_0_)/(S_39_-S_0_)
Washout slope	Slope_wash_	Slope_wash_ indicates the slope of the linear regression equation defining the linear correlation between the number of scans after T_peak_ and the signal intensity (Fig 1)

S_0_, initial signal intensity of the tumor before CA injection; S_1_, signal intensity of the tumor at the first scan post-CA injection; S_39_, signal intensity of the tumor at the last scan post-CA injection; T_1_, time from the start of CA injection to the completion of the first post-DCE sequence(79s); T_39_, time from the start of CA injection to the final completion of the final post-DCE sequence(1181s).

### Statistical analysis

All statistical evaluations were performed using the SPSS v17.0 (Chicago, IL, USA). One-way ANOVA and the Kruskal-Wallis test were used for the comparison of the variables among the groups based on the homogeneity test of the data. The least significant difference(LSD) test was used to compare two groups if the data were homogeneous. Linear regression was used to explore the relationship between the number of scans after the time to peak (T_peak_) and the signal intensity, and to calculate the fitted line. For all analyses, *p*<0.05 was regarded as significant.

## Results

### MR findings of the Walker 256 breast tumors and TIC types of each group

**Fig 2:**
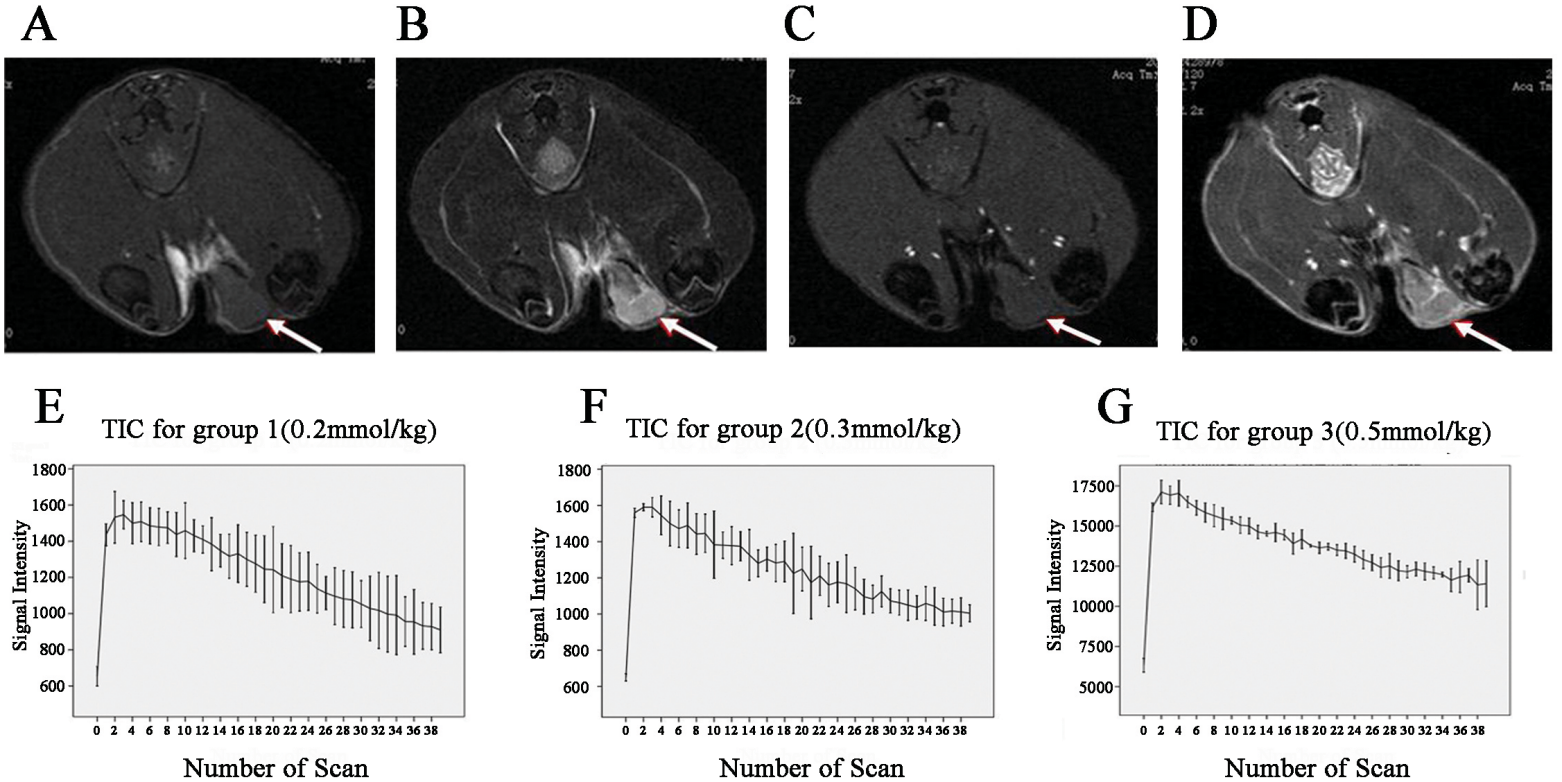
The MRI findings of the Walker 256 breast tumors and TIC types of each group. Murine breast tumor (white arrows) MR findings: isointensity on T1WI (A); hyperintensity or mostly hyperintensity on T2WI (B); isointensity on the pre-contrast DCE images (C); significantly improved signal intensity after the injection of the CA (D). The average signal intensity-time curves of all the three groups were the washout type (type III) (E–G).

The murine breast tumors that we established presented similar MR characteristics to common human breast malignancies, including isointensities on the T1WI, hyperintensities or mostly hyperintensities on the T2WI, and signal intensities significantly improved after the injection of CA (Fig 2A–2D). Regardless of the dosage of CA injected into the rats, all the tumors presented the washout type (type III) TICs (Fig 2E–2G).

### Influence of the contrast agent dosage on the semi-quantitative parameters

**Fig 3:**
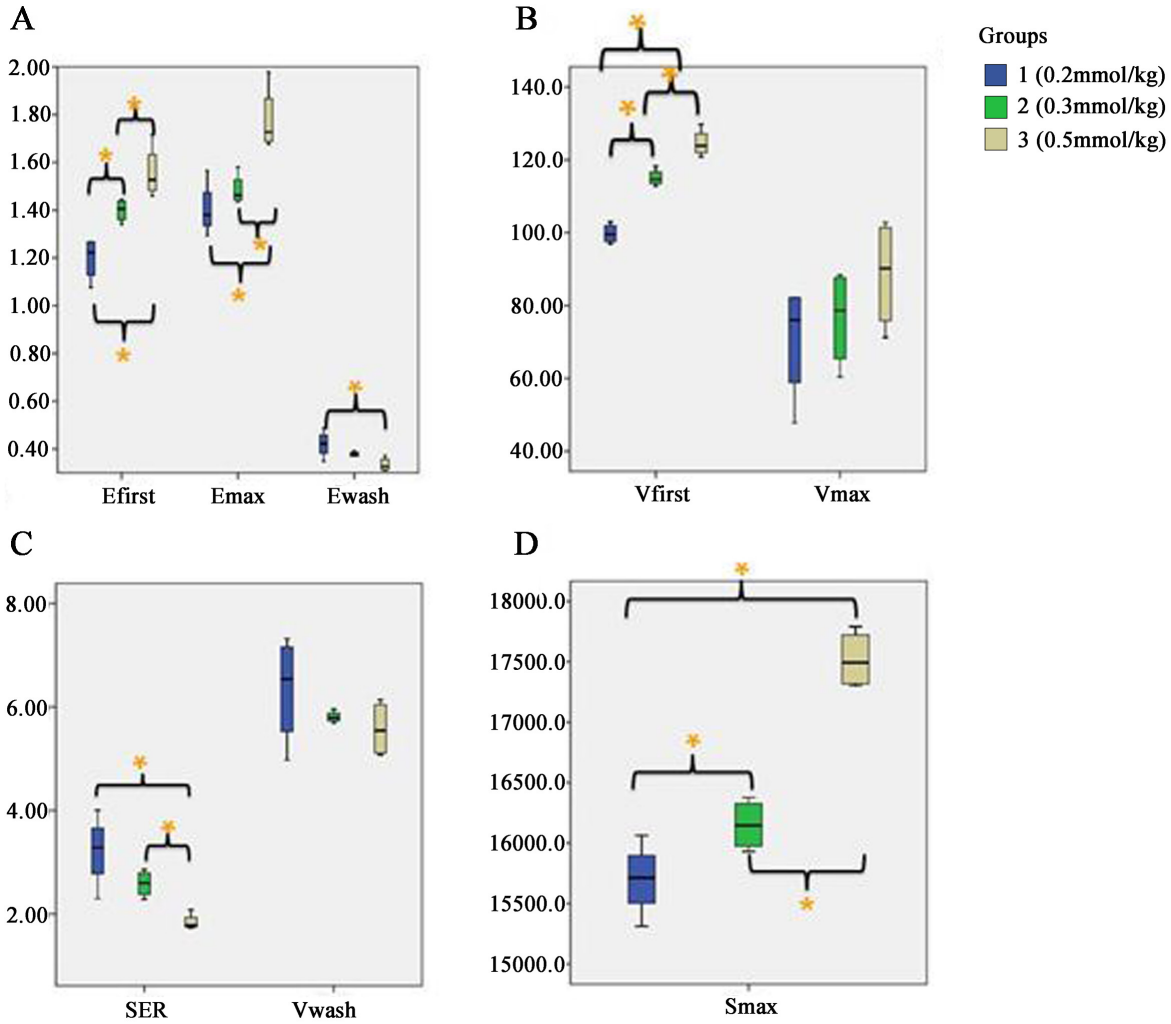
Box plots illustrating the influence of the CA dosage on several semi-quantitative parameters. The results of the pairwise comparisons are also shown in this figure. (A)E_fitst_, E_max_, and E_wash_; (B)V_first_ and V_max_; (C)SER and V_wash_; (D)S_max_. Some parameters that unaffected by the dosage variation, such as Slope_wash_ and Tpeak are not shown. *The difference between two groups is significant (*p*<0.05).

**Fig 4:**
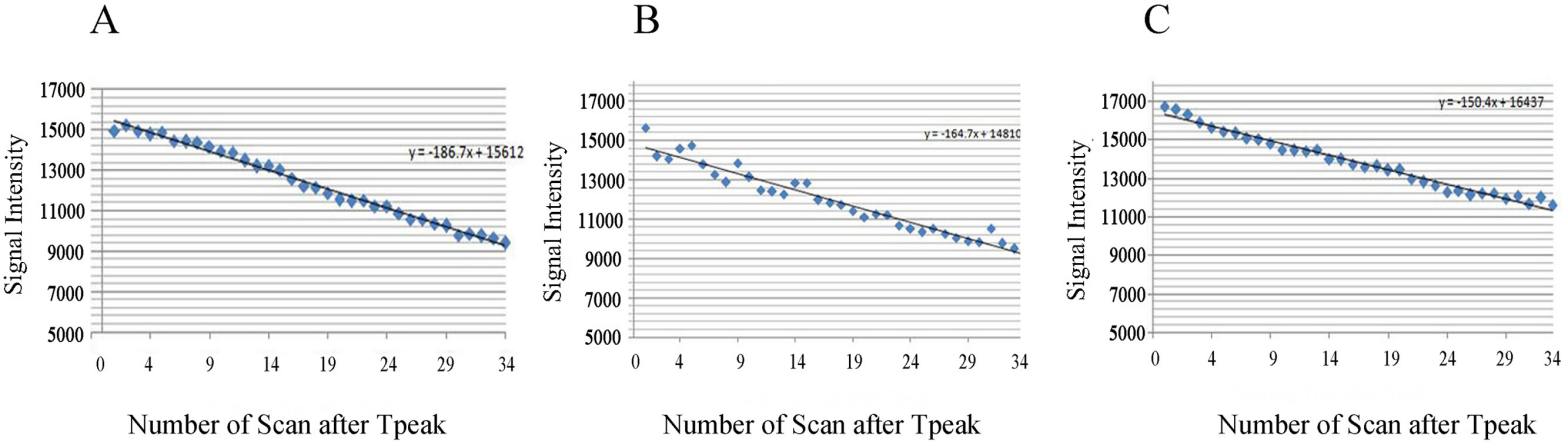
Relationship of the number of scan after T_peak_- with the signal intensity curve for each group. The average signal intensity of each group was linearly correlated with the number of scan after T_peak_. A Group 1;contrast agent (CA) dosage, 0.2mmol/kg; *r* = -0.972;*p*<0.001;linear regression equation, y = -186.777x+15612.354; B Group 2 CA dosage,0.3mmol/kg; *r* = -0.971;*p*<0.001;linear regression equation, y = -164.766x+14809.717; C Group 3;CA dosage,0.5mmol/kg; *r* = -0.989;*p*<0.001; linear regression equation, y = -150.447x+16437.388.

The values of E_first_, V_first_, S_max_, E_max_, E_wash_, and SER differed significantly among the three groups(*F* = 16.952, *p* = 0.001; *F* = 69.483, *p*<0.001; *F* = 54.838, *p*<0.001; *F* = 12.510, *p* = 0.003; *F* = 5.248, *p* = 0.031; and *F* = 9.733, *p* = 0.006, respectively) (Tables 3–5 and Fig 3). However, the T_peak_, V_max_ and V_wash_ values did not differ significantly among the three groups (*F* = 0.065, *p* = 0.937; *F* = 1.505, *p* = 0.273; and *χ2* = 1.423, *p* = 0.319, respectively) (Tables 4 and 5, Fig 3). The mean signal intensities of all three groups presented strongly negative linear correlations with the number of scans after T_peak_ (*r* = -0.972, *p*<0.001; *r* = -0.971, *p*<0.001; *r* = -0.989, *p*<0.001) (Fig 4). The Slope_wash_ values, too, did not differ significantly among the three groups (*F* = 1.654, *p* = 0.244) (Table 5).

**Table 3:** Comparison of theS_0_ and early enhancement parameters among the three dosage groups.

Parameters	CA dosage(mmol/kg)	*x*±*s*	*F*	*p*
S_0_			0.720	0.513
	0.2	6541.874±339.611		
	0.3	6503.018±126.257		
	0.5	6333.638±270.450		
E_first_			16.952	0.001
	0.2	1.197±0.091^a^^b^		
	0.3	1.399±0.048^b^		
	0.5	1.557±0.112		
V_first_			69.483	<0.001
	0.2	99.753±2.717^a^^b^		
	0.3	115.093±2.355 ^b^		
	0.5	124.590±3.768		

One-way ANOVA was used to compare the data among multiple groups and to determine the homogeneity of the data. The least significant difference (LSD) test was used for comparing the variables between two groups. S_0_, initial signal intensity of the tumor before CA injection; E_first_, initial enhancement percentage; V_first_, initial enhancement velocity.

^a^Statistically significant difference when compared to group 2 (dosage,0.3mmol/kg).

^b^Statistically significant difference when compared to group 3 (dosage,0.5mmol/kg).

**Table 4:** Comparison of the peak parameters among the three dosage groups.

Parameters	CA dosage (mmol/kg)	*x*±*s*	*F*	*p*
S_max_			54.838	<0.001
	0.2	15700.428±307.323^a^^b^		
	0.3	16150.568±211.007 ^b^		
	0.5	17519.888±239.868		
T_peak_			0.065	0.937
	0.2	137.000±41.012		
	0.3	129.750±27.765		
	0.5	129.750±27.765		
E_max_			12.510	0.003
	0.2	1.404±0.116 ^b^		
	0.3	1.484±0.066 ^b^		
	0.5	1.777±0.138		
V_max_			1.505	0.273
	0.2	70.497±16.243		
	0.3	76.497±13.487		
	0.5	88.616±15.288		

One-way ANOVA was used for comparison of the data among multiple groups and to determine the homogeneity of the data. The least significant difference(LSD) test was used for comparing the variables between two groups. S_max_, maximum signal; T_peak_, time to peak; E_max_, maximum enhancement percentage; V_max_, maximum enhancement velocity.

^a^Statistically significant difference when compared to group 2 (dosage,0.3mmol/kg).

^b^Statistically significant difference when compared to group 3 (dosage,0.5mmol/kg).

**Table 5:** Comparison of the washout parameters among the three dosage groups.

Parameters	CA dosage (mmol/kg)	*x*±*s/Mean rank*	*F/χ*^*2*^	*p*
E_wash_			5.248	0.031
	0.2	0.420±0.057 ^b^		
	0.3	0.378±0.010		
	0.5	0.377±0.498		
V_wash_			1.423	0.319
	0.2	8.250		
	0.3	5.750		
	0.5	5.500		
SER			9.733	0.006
	0.2	3.216±0.702 ^b^		
	0.3	2.584±0.257 ^b^		
	0.5	1.839±0.162		
Slope_wash_			1.654	0.244
	0.2	-186.778±44.917		
	0.3	-164.766±17.532		
	0.5	-150.448±10.243		

The Kruskal-Wallis test was used to compare the V_wash_ values among the three groups, and one-way ANOVA was used for the comparison of the rest of the parameters. The least significant difference(LSD) test was used for comparing the variables between two groups. E_wash_, washout percentage; V_wash_, washout velocity; SER, signal enhancement ratio; Slope_wash_, washout slope.

^b^Statistically significant difference when compared to group 3 (dosage,0.5mmol/kg).

## Discussion

Nowadays, NAC has been widely used to downstage before surgery in patients with locally advanced breast tumors, aiming to increase the breast conservation rate or to increase the possibility of having surgery for patients who were not initially candidates. Thus, early identification of poor responders is particularly important so that clinicians can change the treatment to a more aggressive option to improve outcome. Various MR imaging techniques, including diffusion weighted imaging (DWI)[22], magnetic resonance spectroscopy imaging (MRS)[23], and particularly DCE-MRI [24] have been used to forecast outcomes in breast cancer patients. DCE-MRI is capable of describing the hemodynamic changes as well as the changes in the tumor size before and after NAC, therefore DCE imaging might help predict the histopathological response to NAC. It is worth noting that DCE imaging data sets are too large and complicated for manual analysis, even with the help of well-designed software. Hence, the use of CAD systems become important in this field.

### The role of quantitative parameters and semi-quantitative parameters in predicting NAC responses

Lots of researchers have reported a great value of quantitative parameters, also known as pharmacokinetic parameters, in predicting the responses to NAC at an early stage. Tateishi et al.[25] reported that the percentage rate constant (%Kep) and percentage area under the time-signal intensity curve over 90 seconds (%AUC_90_) were significant independent predictors of a pathologic complete response (pCR). Additionally, a systematic review [26] based on 605 subjects demonstrated that the reductions in the volume transfer constant (Ktrans) for prediction of the pathologic response. The extra vascular-extracellular volume (Ve) can also be used to monitor the NAC response, as a favorable response to NAC might substantially decrease the tumor cell density, leading to an increase in the Ve [22,27]. However, due to the different pharmacokineticmodels and imaging protocols used for building these parameters [28], discrepancies exist among the various studies reported. Furthermore, the compromised spatial resolution, time consuming nature and expensive software limit the evaluation of the model based parameters. Recently, the model free parameters, also known as semi-quantitative parameters, have drawn attention in predicting the responses to NAC. Several researchers have reported positive results [29–30]. Abramson et al.[24] indicated that the percentage of voxels demonstrating washout kinetics in the tumor after 1 cycle of NAC decreased by64% discriminating the pCR with sensitivity of 100%, and a specificity of 66.67%. Another study pointed out that changes in the curve shape were significantly correlated with the clinical and pathological response (*p* = 0.005, *p* = 0.005, respectively), and changes in the curve shape were significantly related to changes in Ktrans (*p*<0.001)[31], which is a common predictor in monitoring NAC response. Cho et al [32] showed that signal changes in every voxel after 1 cycle of NAC may enable early identification of the pCR, while the pharmacokinetic parameters did not. Yi’s [10] results showed shorter T_peak_ for the tumor was correlated with negative oestrogen receptor status (*p* = 0.037), which is generally considered to be a poor predictor for NAC [33].

### Different scanning protocols lead to inconsistent semi-quantitative parameters

Despite the satisfactory results obtained using semi-quantitative parameters as possible predictors, the complex scanning protocols involving scanners, CA, sequences, etc. can affect the final analysis of the DCE-imaging data. Differences in the delayed post-contrast timing might strongly affect the kinetics assessments for malignancies, as the volume percentage of washout in malignancies increased at 7.5min compared with 4.5min [34]. Despite the fact that there was no significant difference in the dynamic contrast-enhanced kinetics at 3 T compared to 1.5 T, the correlation between the size measurements based on MRI and those based on pathological findings were higher at3 T (*ρ* = 0.66, *p* = 0.002) than at 1.5 T (*ρ* = 0.36, *p* = 0.13)[35]. Although many researchers have studied numerous factors affecting the DCE-imaging data, few studies have focused on the CA dosage. Jansen’s [21] results as mentioned above roughly showed the effect of CA dosage on several semi-quantitative parameters. To determine which parameters are dose-independent, we used murine mammary carcinogenesis models divided into three groups according to the different CA dosages. Since most of the experiments on mouse models in DCE studies have used slightly higher CA doses than conventional human MR examinations [27,36–37] and in order to account for the potential differences in the dosages in human MR examinations, we chose the CA dosages of 0.2 mmol/kg and 0.3 mmol/kg. In order to ensure that the identified parameters that were truly independent of the CA dosage changes, we added an additional group (0.5 mmol/kg) to increase the differences among the dosage groups. We also evaluated greater number of semi-quantitative parameters than the previous studies, aiming to identify stable parameters for further use with CAD systems in breast cancer.

### Dose-dependent semi-quantitative parameters

Our results also showed that the early enhancement parameters (E_first_ and V_first_), several of the peak parameters (S_max_ and E_max_), and the washout parameters (E_wash_ and SER) were affected by the CA dosage. A decrease in the values of E_first_, V_first_, and S_max_ might be used as a sign of effective chemotherapy, as chemotherapy drugs might effectively reduce the microvessel density in the tumor, which, in turn, would reduce the values of E_first_, V_first_, and S_max_. However, our results indicated these parameters were greatly affected by the CA dosage; thus, it was, therefore, important to determine whether different CA dosages were used during the DCE scanning when we assessed these parameters. Although E_max_ also had a certain dose-dependence, there were no differences between the group 1 (0.2 mmol/kg) and group 2 (0.3 mmol/kg). Jansen et al.[21] pointed out that there was a trend for E_max_ to increase when the doses increased, but the differences weren’t significant. The dosage variations evaluated in our research were larger than those reported in Jansen’s research, therefore, our findings might suggest a greater effect of dosage on E_max_. The E_wash_ values might also affected by the CA dosage when there were greater deviations between dosages(comparison of the E_wash_ values obtained with0.2mmol/kg and 0.5mmol/kg CA dosages, *p* = 0.010). SER is a commonly used parameter in diagnosis and to predict the NAC response. SER values also reflect the microvessel density, and ahigher SER in parenchyma around the breast cancer might be associated with a lower recurrence rate [38], while a higher SER in breast cancer was correlated with the absence of a response toNAC [39]. Our research demonstrated a similar effect of dosage on SER as that on E_max_. There were significant differences in the SER values between the0.2mmol/kg and 0.5mmol/kg CA dosage groups and the 0.3 mmol/kg and 0.5mmol/k dosage groups (*p* = 0.002 and *p* = 0.041, respectively);however, there were no significant differences in the SER values between the 0.2 mmol/kg and 0.3mmol/kg dosage groups(*p* = 0.074).

### Dose-independent semi-quantitative parameters

The TIC can reflect the composite of tissue perfusion, vessel permeability, and extravascular-extracellular space, and it has been used in many studies to differentiate the benign and malignant diseases [11] and to predict the NAC responses [9]. Our results demonstrated that the TIC type did not depend on the CA dosage; rather, the TIC type represents the tumor’s histological and biological features. Therefore, the TIC is of great importance for monitoring the response to NAC.

Our study showed that the rest of the peak parameters (T_peak_ and V_max_) and the washout parameters (V_wash_ and Slope_wash_) were dose-independent. After chemotherapy, the T_peak_ value might be reduced due to the decrease in angiogenesis. Our results indicated that T_peak_ didn’t change when the CA dosage changed, which is consistent with the findings reported by Jansen [21]. Additionally, we also found that the V_max_ and V_wash_ values were unaffected by the CA dosage(*p* = 0.237, *p* = 0.319, respectively). The CA infiltration rate tended towards saturation, which was likely related to the result of V_max_. Though V_wash_ didn’t be affected by CA dosage, when we calculated V_wash_, we noticed that there were relatively large individual differences in the T_peak_ values(range, 108s to 195s). However, whether this would affect the results of V_wash_ needs to be confirmed, as(T_39_-T_peak_) value was the denominator in the calculation of V_wash_. Galiè et al.[40] defined a washout slope distinct from the traditionally defined slopes in other studies; according to the authors, the latter slope actually described the velocity or enhancement percentage but not the actual slope [8,10,12]. We defined the Washout_slope_ in our study based on the definition reported by Galiè et al. (Table 2 and Fig 1; with 34 repetitions for all 21 models). Then, we determined whether there were differences in the Washout_slope_ values among the three groups. The results showed that Washout_slope_ was dose-independent (*p* = 0.224), which indicated that the absolute speed of the outflow didn’t change with the dosage; this result corroborated that obtained forV_wash_. However, further clinical trials might be required to verify the role of this newly defined parameter differential diagnosis and monitoring the NAC response. Moreover, the accuracy of the Washout_slope_ values might be more accurate with more delayed phases, because the post-contrast phase number influences the accuracy of the fitting line. However, due to the use of simple and fast scanning protocols, this presents an issue that needs to be addressed in the future.

## Conclusion

In conclusion, despite the small sample size of our experiment, we found that the CA dosage had no effect on a few of the evaluated semi-quantitative DCE parameters. These dose-independent parameters (T_peak_, V_max_, V_wash_, and Slope_wash_), along with the TIC type, might be more meaningful for monitoring the NAC response because they enable the comparison of data regardless of the variation of the CA dosage during DCE scanning. Certainly, The dose-independent parameters might play greater roles in CAD systems than the dose-dependent parameters.
